# Evidence on Serum Anti-Müllerian Hormone Levels and Endometriosis Surgery

**DOI:** 10.3390/jcm14113772

**Published:** 2025-05-28

**Authors:** Georgios Grigoriadis, Angelos Daniilidis, Anna Pitsillidi, Ismail Biyik, Adrien Crestani, Benjamin Merlot, Horace Roman

**Affiliations:** 1 1st University Department in Obstetrics and Gynecology, Papageorgiou General Hospital, School of Medicine, Aristotle University of Thessaloniki, 546 43 Thessaloniki, Greece; angedan@hotmail.com; 2 Rheinland Klinikum Dormagen, Dr.-Geldmacher-Straße 20, 41540 Dormagen, Germany; anna.pitsillidi@gmail.com; 3 Department of Obstetrics and Gynecology, School of Medicine, Kütahya Health Sciences University, 43700 Kütahya, Turkey; dribiyik@hotmail.com; 4 Institut Franco-Europeen Multidisciplinaire d’Endometriose (IFEMEndo), Endometriosis Centre, Clinique Tivoli-Ducos, 33000 Bordeaux, France; adriencrestani@hotmail.fr (A.C.); merlot.benjamin@gmail.com (B.M.); horace.roman@gmail.com (H.R.); 5 Franco-European Multidisciplinary Endometriosis Institute (IFEMEndo), Middle East Clinic, Burjeel Medical City, Abu Dhabi 7400, United Arab Emirates; 6 The Aarhus Endometriosis Center, Department of Obstetrics and Gynecology, Aarhus University Hospital, 8000 Aarhus, Denmark

**Keywords:** endometriosis, endometrioma, deep endometriosis, superficial endometriosis, ovarian reserve, anti-Müllerian hormone, AMH, infertility, reproductive surgery

## Abstract

The assessment of the ovarian reserve is important in patients with fertility intent. The anti-Müllerian hormone (AMH) serum level is a useful ovarian reserve marker. Endometriosis is a benign disease with three phenotypes: superficial peritoneal endometriosis (SUP), ovarian endometrioma (OMA), and deep endometriosis (DE). Endometriosis is linked with infertility; however, the exact impact of endometriosis and endometriosis surgery on AMH levels is less clear. This narrative review examines how different endometriosis phenotypes and related surgeries affect AMH levels as well as explores whether pre- and post-surgical AMH can predict the reproductive outcomes in women seeking pregnancy. The evidence suggests that OMA is linked to reduced AMH values and a higher AMH decline rate over time. OMA cystectomy causes further a reduction in AMH, which, however, tends to recover postoperatively. Non-excisional surgery for OMA spares the ovarian parenchyma; however, an at least temporary decline in AMH is observed. The effect is likely smaller than that of cystectomy. Non-thermal methods of hemostasis following cystectomy are likely superior in terms of AMH. The AMH levels before OMA cystectomy appear to be positively correlated with the postoperative probability of pregnancy, particularly spontaneous conception, but not livebirth rates. Preoperative AMH levels are also predictive of the risk of diminished ovarian reserve (DOR). Similarly, postoperative AMH levels and the rate of AMH decline at 1 year after OMA cystectomy appear to be predictive of fertility outcomes. SUP likely has little (if any) impact on AMH levels. DE reduces AMH levels, and a further reduction following surgery is anticipated. However, a reduction in AMH values should not be interpreted as a decline in the patient’s reproductive potential. Further research should focus on the extra-ovarian locations of endometriosis and their impact on AMH values.

## 1. Introduction

Endometriosis is a common, benign gynecological disease of unknown etiology, affecting 1 in 10 women of reproductive age [1]. It is characterized by the presence of ectopic, endometrium-like tissue, leading to an estrogen-dependent, chronic inflammatory process and is commonly linked with pelvic pain and/or infertility [2]. The fecundity rates in couples of reproductive age with no documented infertility are estimated to range between 15% and 20%, whereas in those with untreated endometriosis, rates vary from 2% to 10% [3]. Three phenotypes of endometriosis are commonly recognized: ovarian endometrioma (OMA), superficial peritoneal endometriosis (SUP), and deep endometriosis (DE) [1].

Anti-Müllerian hormone (AMH) is produced by the granulosa cells of the ovary, playing an important regulatory role in ovarian folliculogenesis and preventing the premature exhaustion of the ovarian reserve [4]. The AMH levels in the blood serum are believed to accurately reflect the ovarian reserve [5], with no significant variations during the menstrual cycle [6], making it a useful marker in daily clinical practice. However, it is important to note that assay-specific differences exist, as various commercial assays may considerably vary in absolute values [7,8].

AMH levels are useful in predicting the response to controlled ovarian stimulation (COS) protocols as part of artificial reproductive technology (ART) techniques [9], as the number of retrieved oocytes may depend on the AMH value [10]. Furthermore, AMH levels progressively decrease with advancing patient age [11], and age-specific AMH values may be useful in predicting age at natural menopause [12]. However, its usefulness in predicting future fecundity and chances of natural conception is limited, based on existing evidence [13,14,15]. However, these studies excluded women with endometriosis [13,14,15].

While there is a well-known link between endometriosis and infertility, there is conflicting evidence regarding the potential impact of various endometriosis phenotypes on serum AMH levels [16,17,18,19,20]. Regarding the effect of endometriosis surgery on AMH values, the majority of the published literature focuses on patients with OMA, raising concerns regarding a potentially harmful effect of surgery on the ovarian reserve [21,22,23]. Furthermore, identifying the precise clinical significance of serum AMH levels pre- and post-surgery in terms of predicting the probability of pregnancy would be most useful in patients with endometriosis with intentions for pregnancy.

In this narrative review, we critically present the available evidence regarding the impact of different endometriosis phenotypes, as well as the effect of endometriosis surgery, on AMH levels. We also discuss if AMH levels pre- and post- endometriosis surgery may be useful in predicting the reproductive outcomes of patients with endometriosis and pregnancy intention.

## 2. Materials and Methods

We performed a literature search for relevant articles in the following databases: PubMed, Embase, Scopus, Web of Science, and Cochrane Library. We used combinations of the following MESH terms and keywords: “endometriosis”, “endometrioma”, “ovarian endometrioma”, “ovarian endometriosis”, “superficial endometriosis”, “deep endometriosis”, “deep infiltrating endometriosis”, “bowel endometriosis”, “colorectal endometriosis”, “bladder endometriosis”, “anti-Müllerian hormone (AMH)”, “ovarian reserve”, and “fertility outcomes”. We included English-language articles only, published between the years 2000 and 2025. We included both original research as well as review articles. Conference abstracts were excluded. The search was performed independently by two authors (G.G., A.P.), who reviewed the abstracts and relevant full texts. A third author (I.B.) was allocated to resolve any disagreements on article selection. No disagreements were noted.

## 3. Results

### 3.1. The Impact of Endometriosis on AMH Levels

It has been well demonstrated that all phenotypes of endometriosis have a negative impact on fertility [24] through a variety of pathophysiological mechanisms, such as altered peritoneal environment, distorted pelvic anatomy, altered endometrial receptivity, impaired implantation, poor oocyte quality, abnormal utero-tubal transport, endocrine abnormalities, cell signaling, and epigenetic changes [25].

Recently published data from the Nurses’ Health Study II (NHS II) identified women with laparoscopically confirmed endometriosis *(n* = 119) to have 29.6% lower AMH levels (95% CI: −45.4, −9.2) compared to those without endometriosis (*n* = 1842), a finding that did not vary based on parity or infertility history [26]. However, the mechanisms responsible for reduced AMH levels in patients with endometriosis, particularly those without OMA, are not entirely clear. Chronic inflammation and oxidative stress due to endometriosis may exert a negative effect on the ovarian reserve [19]. Patients with endometriosis exhibit an increase in the apoptosis of the cumulus cells surrounding the oocyte, in particular the granulosa cells [27,28], with the apoptosis likely being proportional to disease severity [29]. Furthermore, a “burn-out” effect on the follicular pool through continuous activation and depletion of follicles, as seen in cases of chemotherapy-induced gonadotoxicity, may also be present in cases of endometriosis [30], possibly explaining why a diagnosis of endometriosis has been linked with an earlier age of menopause [31,32]. Carrarelli et al. suggested that AMH itself may actually play a role in the pathogenesis of and growth in endometriosis, as they identified increased AMH and AMHRII mRNA and protein expressions in the endometrium of patients with endometriosis compared to controls, as well as in endometriotic lesions, although no significant difference in the serum AMH levels was noted between the groups [33]. Furthermore, the peritoneal fluid of patients with advanced-stage endometriosis exhibited reduced AMH levels compared to controls [34].

With respect to SUP (or minimal/mild endometriosis (MME)), its negative effect on fertility outcomes is well established [35]. However, the potential impact of SUP on ovarian reserve is, to date, less clear. In Lessan’s recent case-control study, the AMH levels in patients with histologically proven SUP and no co-existing OMA or DE did not differ significantly from controls [3.0 ± 2.8 ng/mL and 2.8 ± 1.9 ng/mL, respectively *(p =* 0.71)] [16]. However, controls did not have SUP ruled out via laparoscopy, and, as such, it is possible that some of them might have actually had SUP. Similarly, a prospective cohort study did not identify a significant difference in the baseline AMH levels between those with SUP and endometriosis-free controls (*p* = 0.19) [23]. A case–control study by Shebl et al. identified no significant difference in the serum AMH levels between patients with MME and those undergoing IVF/ICSCI due to male factor infertility [20]. Follicular fluid AMH levels did not differ significantly between those with MME undergoing natural IVF and non-endometriotic controls [36,37]. In contrast to these finding, Lemos et al. identified that patients with infertility and laparoscopically proven MME had lower AMH values (1.26 ± 0.7 ng/mL) compared to patients who were infertile due to tubal occlusion (2.02 ± 0.72 ng/mL, *p* = 0.004) [38]. We need to bear in mind the small sample size (*n* = 34), the inclusion of patients who were infertile only, and that the results were not adjusted to parity or hormonal treatment. The findings of Lemos et al. are supported by a recently published study in a rat model where surgically induced peritoneal endometriosis led to a decline in the measured AMH levels [39].

Regarding the presence of OMA, there is overwhelming evidence showing that it is associated with a reduction in AMH levels compared to controls [17,21,22,23]. The systematic review and meta-analysis of 17 studies by Muzii et al. found that patients with OMA had lower AMH compared to those with other types of benign ovarian cysts (mean difference = −0.85, 95% CI: −1.37 to −0.32) as well as those with healthy ovaries (mean difference = −0.61, 95% CI: −0.99 to −0.24) [19]. An earlier prospective study found patients with OMA to have lower AMH compared with controls at baseline (4.2 + 2.3 versus 2.8 + 2.2 ng/mL, respectively, *p* = 0.02) [22]. Another prospective study confirmed the above finding (baseline AMH values in the OMA group = 1.8 ng/mL, 95% CI: 1.2–2.4 ng/mL vs. negative laparoscopy group = 3.2 ng/mL, 95% CI: 2.0–4.4 ng/mL; *p* < 0.02) [25]. Furthermore, patients with OMA tend to have a more rapid decline in AMH values over time compared to controls [40,41]. The bilaterality of OMA has also been demonstrated to exhibit a negative effect on AMH values compared to unilateral OMA [39,41].

Regarding DE, the existing evidence suggests a detrimental effect on AMH levels [16,17,18]. Pacchiarotti et al. demonstrated that patients with stage III/IV endometriosis had significantly lower AMH levels (0.97 ± 0.59 ng/mL) compared to presumed endometriosis-free, age-matched controls (1.72 ± 0.63 ng/mL, *p* = 0.001) [19]; however, the authors did not clarify what percentage of patients with DE had co-existing OMA, while controls did not have a diagnosis of endometriosis ruled out by laparoscopy. A case–control study reported that patients with stage III/IV endometriosis had significantly lower AMH levels (2.38 ± 1.83 ng/mL) compared to controls undergoing IVF/ICSI due to male factor (3.58 ± 2.46 ng/mL, *p* < 0.0001), whereas there was no statistically significant difference between those with stage I/II disease and controls [20]. The same study identified a significant difference when comparing AMH levels between patients with stage I/II and stage III/IV endometriosis (stage Ι/ΙΙ: 3.28 ± 1.93 ng/mL vs. stage III/IV: 2.38 ± 1.83 ng/mL, *p* < 0.01) [20]. While controls were matched for age, frequency of infertility, and body mass index (BMI), the authors did not clarify the percentage of patients with stage III/IV endometriosis who also had OMA. These findings may be viewed as anticipated, since superior postoperative fertility was demonstrated in milder (stage I/II) cases compared to more advanced (stage III/IV) endometriosis [42]. In Kim et al.’s retrospective case–control study comparing AMH levels between patients with OMA and age-matched controls without ovarian cyst, multiples of the median for AMH (AMH-MoM) levels were negatively correlated with the estimated endometriosis score (r^2^ = 0.13, *p* < 0.01) [43]; in particular, patients with rASRM stage IV endometriosis and OMA had significantly lower AMH and AMH-MoM compared to controls (2.1 ± 0.3 vs. 3.1 ± 0.4 ng/mL, *p* = 0.02 and 1.1 ± 0.1 vs. 1.7 ± 0.2, *p* = 0.03, respectively), but there was no statistically significant difference in terms of AMH or AMH-MoM when patients with stage III endometriosis and OMA were compared to controls (3.7 ± 0.5 vs. 3.4 ± 0.5 ng/mL and 1.6 ± 0.2 vs. 1.5 ± 0.2, respectively) [43]. In their case–control study, Streuli et al. did not identify a significant difference in the AMH levels between cases (various endometriosis phenotypes and disease stages) and controls (other, benign gynecological pathologies) [cases: 3.6 ± 3.1 ng/mL, controls: 4.1 ± 3.4 ng/mL, mean difference: 0.45 (95% CI −0.02 to 0.96) ng/mL, *p* = 0.06], with the only exception being patients with previously operated OMA [44].

### 3.2. The Impact of Endometriosis Surgery on AMH Levels

The majority of published evidence focuses on patients undergoing surgery for OMA. The optimal method of surgical management of OMA is still debated, with different approaches including ovarian cystectomy, ablative techniques (laser, plasma energy or bipolar diathermy), ethanol sclerotherapy, and combined approaches [21]. Cystectomy has been linked to reduced risks of OMA recurrence and endometriosis-associated pain [45], as well as increased chances of spontaneous conception [46]. However, it may reduce ovarian volume [47], while it presents an estimated risk of ovarian failure of 2.4% [48]. These concerns have led to the development of the aforementioned non-excisional alternatives.

A detrimental effect of OMA cystectomy on AMH levels has been demonstrated [49,50], with various studies suggesting that the decline is greater when compared to cystectomy for other benign cysts [51,52,53]. The effect is more profound in cases of bilateral OMAs [54,55]. A systematic review and meta-analysis reported AMH drops of 39.5% and 57.0% for unilateral and bilateral OMA cystectomy, respectively, compared to baseline [54]. The decline may be viewed as a result of the loss of healthy follicles, although a direct association between the number of follicles inadvertently removed and the extent of AMH reduction has not been proven [56]. Cystectomy for bilateral OMAs has been linked with an earlier age of menopause and an increased risk of premature ovarian failure (POF) [57].

Other factors that affect post-cystectomy AMH reduction include the preoperative AMH value [22,58,59,60,61], advancing patient age [59], the duration of surgery [56], and the cyst size [58,62], although a recent meta-analysis of seven prospective studies did not identify significant differences on the AMH values after surgery depending on cyst size [63]. With regard to endometriosis severity, a prospective study found that, although there was no significant correlation between the preoperative AMH levels and the rASRM score (r = −0.219, *p* = 0.187), a positive correlation between the rASRM score and the rate of AMH decline post-OMA cystectomy was noted (r = 0.473, *p* = 0.00273), with patients with more advanced disease experiencing a larger loss of their ovarian reserve [64].

Various systematic reviews and meta-analyses have reported that the use of diathermy for hemostasis following cystectomy has a deleterious effect on AMH values [65,66,67]. Ata at al. found in a meta-analysis of four studies (213 patients) that sutures or hemostatic sealants caused a 6.95% less reduction in AMH at 3 months after cystectomy compared with bipolar diathermy [65]. Another meta-analysis of three studies, despite significant heterogeneity, identified bipolar electrocoagulation as causing a significantly higher AMH decline at 3 months after surgery compared to non-thermal hemostasis methods (mean difference: −0.79 ng/mL, 95% confidence interval: −1.19 to −0.39) [66]. The detrimental effect of bipolar electrocoagulation was maintained at 12 months after surgery, according to a meta-analysis by Ding et al. (weighted mean difference: −1.01 ng/mL; 95% confidence interval: −1.85 to −0.17) [67]. This effect may be unique to OMA cystectomy and not apply to cystectomy for other benign cysts [68]. In contrast to the above, a recent randomized controlled trial (RCT) found no significant differences in the AMH values 6 months after cystectomy for unilateral OMA between bipolar coagulation, hemostatic sealants, and sutures [69]. Similarly, an earlier RCT found no significant difference in AMH values, 3- months after OMA cystectomy between bipolar coagulation and the use of hemostatic sealant, although the latter led to higher antral follicular count (AFC) value postoperatively [70].

In addition to primary OMA cystectomy, the effect of surgical intervention on ovarian reserve has also been explored in cases of recurrent OMA, where the preservation of AMH levels poses an even greater concern. Cystectomy for recurrent OMA is a technically challenging procedure. A case–control study of 36 patients linked cystectomy in this scenario with a significant reduction in AMH (AMH before second surgery: 2.7 ± 1.9 ng/mL, versus AMH after second surgery: 1.2 ± 1.2 ng/mL, *p* < 0.001), although the AMH values did not differ significantly between unoperated cases (AMH: 2.7 ± 1.9 ng/mL) and controls (AMH: 3.1 ± 1.9 ng/mL, *p* = 0.59) [71]. Therefore, the authors concluded that recurrent OMA does not cause a reduction in AMH values unless surgery is performed [69]. In this setting, ethanol sclerotherapy may be of use, as a retrospective study showed no differences in the AMH decline rates between primary and recurrent OMA managed with this technique [72].

Despite concerns with surgery-induced decreases in ovarian reserves, various studies have suggested a progressive recovery in AMH values postoperatively [73,74,75,76,77]. In a prospective study of 39 patients, 20 of them had higher AMH levels 1 year compared to 1 month after OMA cystectomy [73]. Of note, the follicular loss during surgery was higher (*p* = 0.035) for this group, suggesting that mechanisms other than follicular loss are involved in cases of sustained reduction in AMH post-surgery [73]. Another prospective study with 171 patients showed that, at 12 months after cystectomy, the AMH levels did not differ significantly from the preoperative values in OMAs ≤ 7 centimeters, unilateral cysts, and stage III endometriosis [74]. Two further prospective studies have reported non-significant differences in the AMH values 12 months post-cystectomy compared to baseline [76,77]. This recovery appears less likely in the case of bilateral OMAs [78]. Recovery is likely a result of inflammation-mediated injury triggered by the surgical procedure, which is then followed by the recruitment of and growth in primordial follicles and granulosa cell activation, resulting in a reorganization of the follicular cohort, including follicles transitioning from the “silent” to the “active” state, thus increasing the production of AMH [74]. The estimated 180-day duration of folliculogenesis may explain the delay in AMH recovery observed after surgery [78].

Non-excisional surgical techniques for OMA spare the ovarian parenchyma; therefore, one would anticipate a minimal effect on AMH values. However, the published evidence suggests a temporary, yet significant, detrimental impact on AMH values. A recent prospective study evaluating dual-wavelength laser system (DWLS) diode laser use for endometrioma ablation reported a significant reduction in AMH levels at 3 months compared to baseline (*p* = 0.034). However, at 6 and 12 months, the values were not significantly lower than at baseline [79]. Similarly, an earlier prospective study reported a significant decrease in AMH levels 3 months after plasma energy ablation of OMA, followed by an increase >6 months. No significant difference was noted between pr-operative and postoperative levels at the end of follow-up [80]. Laparoscopic 95% ethanol sclerotherapy of OMA also led to a significant decrease in AMH values [81].

Various comparative studies have found that cystectomy causes a greater depression in AMH values compared to non-excisional approaches to OMA [82,83,84]. Saito et al. reported that bilateral cystectomy for endometriomas led to a significantly higher drop in AMH up to 12 months after surgery compared to bipolar coagulation (*p* = 0.02) [85]. However, no significant difference between the approaches was seen in the case of unilateral OMA, although the AMH values were significantly reduced in both approaches when compared with pre-operative levels [85]. In cases of unilateral OMA < 5 centimeters in diameter, the AMH values at 3 months were comparable between cystectomy and bipolar coagulation; however, for OMA > 5 centimeters, the AMH decline was significantly higher in the cystectomy group [86]. A multi-center RCT found cystectomy to cause a significant decrease in AMH levels at 3 months (from 2.6 ± 1.4 to 1.8 ± 0.8 ng/mL; 95% CI: −1.3 to −0.2; *p* = 0.012), whereas the decline caused by a CO2 laser, compared to baseline, was not significant (from 2.3 ± 1.1 to 1.9 ± 0.9 ng/mL; 95% CI: −1 to −0.2; *p* = 0.09) [83]. A small RCT compared OMA cystectomy (Group 1, *n* = 10) with the “-step procedure” (Group 2, *n* = 10) (laparoscopic drainage of the OMA, followed by 3 months of gonadotrophin-releasing hormone agonists, and then ablation of the cyst wall) [85]. The authors observed a significant decline in Group 1 AMH (from 3.9 ± 0.4 to 2.9 ± 0.2 ng/mL, *p* = 0.026), whereas no significant change was noted in Group 2 (from 4.5 ± 0.4 to 3.99 ± 0.6 ng/mL, *p* > 0.05) [84]. A recent meta-analysis, however, found no significant difference between cystectomy and ablative OMA approaches in terms of AMH decline [87].

The phase of the menstrual cycle at which OMA cystectomy takes place influences the degree of AMH decline inflicted by surgery, according to a recent RCT [88]. The serum AMH values at six months postoperatively in the “late luteal phase” (“LLP”) group was significantly higher than that in the “early follicular phase” (“EFP”) group (3.35 ± 1.67 vs. 2.61 ± 1.15, *p* = 0.018) [86]. Furthermore, the decreases in AMH values six months postoperatively were significantly higher in the “EFP” group than that in the “LLP” group (1.54 ± 0.93 vs. 1.91 ± 1.06; *p* < 0.001) [88]. However, the above findings were not confirmed by a prospective cohort study [89]. The rate of AMH decline at 6 months post-cystectomy did not differ based on the phase of the menstrual cycle in which the cystectomy was performed (follicular phase: 24.5% versus luteal phase: 19.5%, *p* > 0.05) [89].

Robotic surgery (or robot-assisted laparoscopy) has recently been gaining popularity in the surgical management of endometriosis [90]. However, comparative data focusing on whether it confers any benefit over conventional laparoscopy, in terms of AMH values, are scarce. A recently published, retrospective study found no significant differences in the AMH values between robotic and laparoscopic OMA cystectomy [91]. Another retrospective study comparing single-port, robot-assisted laparoscopy versus single-port laparoscopy found the former approach linked to higher AMH values after the procedure; however, this was only the case for cases of stage I/II endometriosis [92].

The evidence on the impact of surgery for extra-ovarian localizations of endometriosis on the AMH values is limited. Regarding SUP, the AMH levels among endometriosis-free controls and those that had superficial disease excised laparoscopically did not differ at the 1-month (*p* = 0.16) or 6-month (*p* = 0.59) follow-up after excision, suggesting that surgery for this endometriosis phenotype does not have a deleterious effect on ovarian reserve [23]. The larger AMH decline following OMA cystectomy when this co-exists with DE [55] may be attributed to a reduction in ovarian blood flow as a result of the extensive adhesiolysis required at the time of surgery. A recent cross-sectional study reported that, when OMA co-exists with DE, surgery results in a higher drop in the AMH compared to OMA alone or DE alone [93].

### 3.3. AMH Levels as Predictor of Fertility Outcomes After Endometriosis Surgery

The latest European Society of Human Reproduction and Embryology (ESHRE) guideline on endometriosis suggests that the patient’s ovarian reserve should be taken into account when considering surgery to enhance chances of natural conception [45]. The precise clinical significance of AMH levels, in terms of predicting the probability of pregnancy after endometriosis surgery, is an interesting topic that, however, needs to be further investigated. Stochino-Loi et al. used an AMH cut-off value of 2 ng/mL, with patients having levels ≥2 ng/mL considered as “normal AMH” levels and those with values <2 ng/mL as “low AMH” levels [94]. They further classified those with levels <1 ng/mL into the “very low AMH” group. Similarly, in a prospective cohort study of patients planning to undergo ovarian cystectomy for OMA, patients were grouped in a “high AMH” group (AMH > 2 ng/mL) and a “low AMH” group (AMH ≤ 2 ng/mL) [95]. The AMH threshold value of 2 ng/mL was previously suggested by Reichman et al. [96]; however, this was based on the cancellation rates of IVF cycles and was not restricted to patients with endometriosis.

Concrete evidence on an optimal preoperative AMH value to predict postoperative fecundity is currently lacking. Zhou et al.’s prospective study included 103 patients with OMA that underwent laparoscopic cystectomy [95]. They observed that the cumulative pregnancy rate (CPR) during the 2-year follow-up after surgery was significantly higher in the “high AMH” group (AMH > 2 ng/mL, *n* = 61) compared to the “low AMH” group (AMH ≤ 2 ng/mL, *n* = 42) (*p* < 0.001), suggesting that a high preoperative AMH is a strong predictor of spontaneous conception after OMA cystectomy. Specifically, the likelihood of conception during the 12–24 months postoperation was 50.82% and 69.44% in the “high AMH” group, while the CPR was 28.57% and 33.61% in the “low AMH” group. They identified the optimal preoperative AMH value as being 3.545 ng/mL. The AMH values dropped significantly in both groups after surgery, but the decline was significantly higher in the “low AMH” group (*p* < 0.001). Another observational study proposed a very similar optimal AMH value of 3.68 ng/mL before surgery as being linked with increased spontaneous conception rates after OMA cystectomy [97].

The surgical management of OMA is not routinely recommended before IVF, as no clear benefit has been demonstrated and surgery may reduce the ovarian reserve [45]. However, if infertility treatments are eventually required after laparoscopic OMA cystectomy, the AMH levels at 1 year after surgery are higher in patients who are pregnant compared to their non-pregnant counterparts, despite no significant differences in the values before or at 1 month after surgery between the groups [98]. Furthermore, not only the AMH value but also the rate of AMH decline after surgery is of importance, as those that conceived spontaneously had a lower rate of AMH decline at 1 year postoperation compared to those that required infertility treatments [53]. A progressively declining AMH value after cystectomy may therefore be viewed as an indication for earlier referral to IVF, in light of these findings.

The preoperative AMH levels may be used to predict the risk of diminished ovarian reserve (DOR) (AMH < 1.1 ng/mL) after laparoscopic OMA cystectomy, according to the Bologna criteria by the ESHRE [99]. For unilateral cystectomy, a preoperative AMH cut-off value of 2.1 ng/mL was predictive of DOR, whereas, for bilateral cystectomy, the cut-off value was 3.0 ng/mL [100]. Bilaterality of OMA was predictive of postoperative DOR. This finding is of clinical importance in the ART setting, as patients with postoperative DOR may be poor ovarian responders and be linked to lower clinical and livebirth rates following IVF compared to idiopathic poor responders [101]. Furthermore, the authors observed that the cumulative spontaneous pregnancy rate of the DOR group was significantly lower than that of the non-DOR group (14.3% vs. 59.2%, respectively, *p* = 0.04) [100].

The surgical management of DE has been demonstrated to enhance fecundity in appropriately selected cases [102,103]. In a retrospective study of 118 patients, Arfi et al. operated for DE without bowel involvement and with pregnancy intention; an AMH level >2 ng/mL was a predictive factor of obtaining spontaneous conception postoperation compared to pregnancy through ART (*p* = 0.0006), although it was not predictive of a live birth [104]. It is worth noting that 36 patients (30.6%) required cystectomy for concurrent OMA. Another retrospective study that focused on the laparoscopic management of bladder DE identified that the only patients with pregnancy intention that failed to conceive after surgery were those with documented DOR preoperation [105]; however, the actual number of patients (nine patients with pregnancy intention, five spontaneous conceptions, and one conception through IVF) was too small to allow for meaningful conclusions [105]. Conversely, another retrospective study did not identify preoperative AMH values to be predictive of conception (natural or through IVF) after surgery in patients with stage III and IV endometriosis, including cases operated on for colorectal endometriosis, with a mean age of 30 years [94]. Conception rates, livebirth rates and probability of pregnancy at 12 and 24 months did not differ significantly between those with normal, low, or very low AMH, with the majority of conceptions being spontaneous. Based on those findings, surgery may be viewed as a valid option for young patients with severe endometriosis and a low AMH in whom the results of IVF are anticipated to be suboptimal. However, the authors recognized that the small number of patients with low or very low AMH (*n* = 46), in comparison to those with normal AMH (*n* = 134), may have impacted the interpretation of the results, as increasing the sample size in the former group could have led to a reduction in the livebirth rate compared to the normal AMH group. First-line surgery has recently been linked to improved pregnancy and livebirth rates compared to first-line IVF in infertile patients with DE without bowel involvement and low AMH (<2 ng/mL) [106].

A recent retrospective study confirmed that, for patients with endometriosis-related infertility, a low preoperative AMH value was negatively associated with chances of conception after laparoscopic surgery; however, it did not affect livebirth rates [42]. According to the authors, this finding is a reflection of the significance of AMH as an ovarian reserve marker but not a marker of the quality of the follicle.

## 4. Conclusions

OMA is consistently associated with decreased serum AMH levels and an accelerated rate of AMH decline over time, even in the absence of surgical intervention. Surgical management, particularly cystectomy, seems to result in a more substantial reduction in AMH, with bilateral procedures and repeated surgeries posing a higher risk to the ovarian reserve. Although partial postoperative recovery of AMH levels may occur, particularly in unilateral cases, this is not uniformly observed and appears less likely in advanced disease. Non-excisional surgical techniques and non-thermal methods of hemostasis may better preserve ovarian function, though current randomized evidence does not demonstrate a definitive advantage.

Pre- and postoperative AMH levels, along with the trajectory in AMH decline, have shown potential for predicting spontaneous conception but have not yet been validated as predictors of livebirth outcomes. AMH is also useful in identifying patients at risk of diminished ovarian reserve following OMA cystectomy, particularly in the context of assisted reproductive technologies.

Evidence regarding extra-ovarian endometriosis remains limited. While SUP appears to exert a minimal influence on AMH levels, DE is associated with reduced AMH, both from the disease process and its surgical treatment. However, the AMH values in DE cases have not been shown to reliably predict fertility outcomes.

Importantly, a reduction in AMH following endometriosis surgery should not be equated with a definitive loss of fertility potential. Future research on AMH in endometriosis should prioritize high-quality, long-term, prospective studies with standardized assays and appropriate control groups to enhance comparability and clinical relevance. RCTs comparing surgical techniques are essential to clarify their impact on ovarian reserve over time. Studies should also stratify patients by disease phenotype and clinical variables, while linking AMH trends to meaningful reproductive outcomes such as time to pregnancy or ART success. Importantly, further research is needed to elucidate the role of AMH in guiding fertility counseling and treatment planning, especially in non-OMA phenotypes and extra-ovarian endometriosis.

## Abbreviations

The following abbreviations are used in this manuscript:

AFC	Antral follicular count
AMH	Anti-Müllerian hormone
ART	Artificial reproductive technology
BMI	Body mass index
CPR	Cumulative pregnancy rate
DE	Deep endometriosis
DOR	Diminished ovarian reserve
DWLS	Dual-wavelength laser system
ICSI	Intracytoplasmic sperm injection
IVF	In vitro fertilization
OMA	Ovarian endometrioma
POF	Premature ovarian failure
RCT	Randomized controlled trial
SUP	Superficial peritoneal endometriosis
